# An MFS Transporter-Like ORF from MDR *Acinetobacter baumannii* AIIMS 7 Is Associated with Adherence and Biofilm Formation on Biotic/Abiotic Surface

**DOI:** 10.1155/2012/490647

**Published:** 2012-02-20

**Authors:** Praveen K. Sahu, Pavithra S. Iyer, Madhumita B. Gaikwad, Sheetal C. Talreja, Karishma R. Pardesi, Balu A. Chopade

**Affiliations:** ^1^Institute of Bioinformatics and Biotechnology, University of Pune, Pune 411007, India; ^2^Department of Microbiology, University of Pune, Pune 411007, India

## Abstract

A major facilitator superfamily (MFS) transporter-like open reading frame (ORF) of 453 bp was identified in a pathogenic strain *Acinetobacter baumannii* AIIMS 7, and its association with adherence and biofilm formation was investigated. Reverse transcription PCR (RT-PCR) showed differential expression in surface-attached biofilm cells than nonadherent cells. *In vitro* translation showed synthesis of a ~17 kDa protein, further confirmed by cloning and heterologous expression in *E. coli* DH5*α*. Up to 2.1-, 3.1-, and 4.1- fold biofilm augmentation was observed on abiotic (polystyrene) and biotic (*S. cerevisiae*/HeLa) surface, respectively. Scanning electron microscopy (SEM) and *gfp*-tagged fluorescence microscopy revealed increased adherence to abiotic (glass) and biotic (*S. cerevisiae*) surface. Extracellular DNA(eDNA) was found significantly during active growth; due to probable involvement of the protein in DNA export, strong sequence homology with MFS transporter proteins, and presence of transmembrane helices. In summary, our findings show that the putative MFS transporter-like ORF (*pmt*) is associated with adherence, biofilm formation, and probable eDNA release in *A. baumannii* AIIMS 7.

## 1. Introduction

Multidrug resistant (MDR) *Acinetobacter baumannii* [1–3] causes a range of infections in hospital environments [4, 5] which are often difficult to treat. Its pathogenesis and resistance are enhanced largely due to formation of biofilms; especially on clinically important surfaces [6]. *Acinetobacter* is capable of surviving on nutrient-limited abiotic surfaces and stressful environmental conditions by forming biofilms [7]. Biofilm formation is a dynamic process involving the attachment of bacteria to a biotic or abiotic surface followed by growth and maturation [8]. Property of adhesion to surfaces and subsequent formation of biofilm can be conferred by large number of genetic factors in MDR *A. baumannii* which are still not completely understood. Biofilm development in *A. baumannii* on abiotic surfaces is mediated by biofilm associated protein-Bap, as shown earlier by Loehfelm et al. [9]. Study by Tomaras and group [10] shows that BfmR, a part of a two-component regulatory system, plays an important role in the morphology of *A. baumannii* 19606 and the ability to form biofilms on abiotic surfaces. Earlier it was shown that expression of *csuAB* gene cluster is required for pili formation and attachment to abiotic surfaces, ensuing biofilm formation [11]. Adherence of *A. baumannii* to polystyrene and respiratory epithelial cells is correlated to antibiotic resistance [12]. 

Transporter proteins in bacteria have been known to have role in biofilm formation as per several recent reports [13–15]. In *E. coli *K-12, a putative transport protein YdgG (TqsA) enhances quorum sensing signal autoinducer 2 (AI-2) secretion or inhibits AI-2 uptake; thereby controlling the overall biofilm formation [13]. In uropathogenic *E. coli, *an autotransporter protein UpaH contributes to biofilm formation and cellular colonization on bladders as shown in a recent study [14]. Similarly, out of an array of proteins identified in nontypeable *H. influenzae* for role in biofilm formation, majority was found involved in cell motility and transport functions such as secretion [15]. In *A. baumannii*, porins, multidrug efflux-pump transport proteins [16] have been a key reason for its emergence as a challenging MDR pathogen in recent times. Besides, in members of *Acinetobacter* group, several transporter proteins are known to date, albeit the functions remained diverse, namely, transport of ions, solutes, and metabolites [17], active efflux of drug molecules [16, 18], uptake and translocation of nucleic acids [19, 20] during natural transformation, and so on. However, function of transport proteins in virulence attributes as biofilms has never been looked into.

It is presumed that transporter-like proteins could have compensatory function to natural transformation seen in members of *Acinetobacter baylyi, Acinetobacter calcoaceticus, *and* Acinetobacter *sp.BD413; which are naturally competent, but not in *Acinetobacter baumannii,* which has evolved from these close genomic species as a pathogen and genetically diversified subsequently [21]. We hypothesize that *A. baumannii,* a strong biofilm former and nosocomial pathogen, may possess transporter-like proteins with multiple functions in biofilm formation. It may also possibly account for eDNA release as demonstrated in our recent work [22] in a pathogenic MDR strain of *A. baumannii, *isolated from ICU, also capable of forming biofilms. The present study was intended to investigate a putative MFS transporter-like ORF (*pmt*) and its association with adherence and biofilm formation on various clinically important surfaces.

## 2. Materials and Methods

### 2.1. Bacterial Strains and Plasmids

A strain of *Acinetobacter baumannii* AIIMS 7 isolated from the bloodstream of a patient in neurosurgery ward of All India Institute of Medical Sciences (AIIMS), New Delhi, India, was used in the study after identification by API 32 GN system [23] and *16SrRNA* gene sequencing (Genbank EU779829). *E. coli* DH5*α* was used as host for the cloning and heterologous expression experiments. Bacterial cultures were maintained on Luria agar and Cysteine Lactose Electrolyte Deficient (CLED) Agar (HiMedia, India). *E. coli* DH5*α*-pGEpmt, *E. coli* DH5*α* pGFP, *E. coli* DH5*α*-pGFP: pGEpmt, and *E. coli* DH5*α*-pGFP: pGEM clones were maintained on Luria agar plates containing 100 mg/L ampicillin. Bacterial strains and plasmids used in the study are listed in Table 1. All bacterial strains were grown at 37°C, 150 rpm with appropriate antibiotic as and when required.

### 2.2. DNA and RNA Manipulations

#### 2.2.1. Nucleic Acid Purification

Genomic DNA was isolated using a commercial kit as per manufacturer's instructions (Sigma Aldrich, USA). Total RNA isolation was done using a total RNA Extraction Kit (Bangalore GeNei, India). Recombinant plasmid purification was done using GenElute Plasmid Miniprep kit (Sigma Aldrich, USA). Nucleic acid samples were quantitatively analyzed in a Biophotometer plus (Eppendorf, Germany) along with purity. DNA samples were size fractionated by agarose gel electrophoresis. 

#### 2.2.2. *In Vitro * Amplification

Primers used to amplify the putative MFS transporter-like ORF (*pmt*) are as indicated in Table 2 (pmt-F and pmt-R). From *A. baumannii *AIIMS 7 genomic DNA, a 735 nt region (containing the *pmt* ORF) was amplified using an optimized PCR program with annealing temperature 45.1°C and annexed with poly-A tailing program. The amplicon was purified using a gel extraction kit (Sigma Aldrich, USA). Amplification was also attempted using plasmid DNA of *A. baumannii* AIIMS 7. PCR reagents and primers were sourced from Sigma-Aldrich. DNase, RNase, and protease-free water was used as negative PCR control in all PCR assays. 

#### 2.2.3. DNA Sequencing and Analysis of Protein Sequence

The purified fragment was sequenced in a 3730 DNA Analyzer (Applied Biosystems, USA) using Big Dye Terminator (BDT) v 3.1 Cycle sequencing reactions. Purification of cycle-sequenced products was done using a BDT X-Terminator Kit (Applied Biosystems, USA) and subjected to capillary electrophoresis in the 3730 DNA Analyzer. Sequence data was processed using Sequencing Analysis Software version 5.1.1 (Applied Biosystems, USA). Sequencing was performed in triplicates. DNA sequences were used for homology studies and *in silico* analysis of the deduced protein using BLAST (NCBI) and ExPASy tools (Swiss Institute of Bioinformatics). Prediction of transmembrane helices in proteins was performed using TMHMM Server v. 2.0 (http://www.cbs.dtu.dk/services/TMHMM/), and signature sequence was predicted using online tool PROSITE (http://prosite.expasy.org/).

### 2.3. Cloning of *pmt*

#### 2.3.1. Ligation and Cloning of Plasmid pGEpmt into *E. coli* DH5*α*

 Purified amplicon was ligated into pGEMT-Easy (3018 bp, Promega, USA). The resulting plasmid designated pGEpmt was transformed into competent *E. coli* DH5*α* [24]. Transformed colonies were selected on LB-Amp plates at 37°C overnight. 

#### 2.3.2. Confirmation of Clones

 Individual antibiotic resistant colonies of recombinant *E. coli* DH5*α* containing the *pmt* gene (*E. coli* DH5*α* pGEpmt) were confirmed for presence of *pmt *using PCR primers. 

### 2.4. Transcriptional Analysis of *Pmt*

#### 2.4.1. Transcription Analysis

 Expression of *pmt* was tested by RT-PCR analysis using total RNA isolated from bacteria grown in LB broth. The RNA samples were treated with RNase-free DNase I (Sigma Aldrich, USA). RT-PCR was carried out using an RT-PCR kit (Invitrogen, USA), as per the manufacturer's instructions and using *16SrRNA* as a control. Direct-PCR of total RNA without reverse transcription was used to test for DNA contamination of RNA samples. Primers pmt RT-F and pmt RT-R were used for amplification of an internal *pmt* region. The amplicons were analyzed by agarose gel electrophoresis using the Tris-borate/EDTA buffer system and ethidium bromide staining. The sequence identity of the amplicons was confirmed by standard automated capillary DNA sequencing as described above.

#### 2.4.2. Expressional Difference

 Variation of gene expression in planktonic and biofilm mode of growth was checked by RT-PCR, as per the methods described above. Overnight grown *Acinetobacter baumannii* AIIMS 7 was diluted 1 : 100 with sterile LB broth in 6-well polystyrene culture plates (Tarsons, India) and were incubated for 24 hours at 37°C static. The plate was sonicated moderately to remove loosely adherent cells at 53 KHz, 2 mins at 25°C in a Sonicator waterbath (Equitron, India), followed by washing twice with phosphate buffered saline. Adherent cells were gently scraped from the culture plate bottom, and total RNA was purified as described above. For planktonic cells, the total RNA was purified from overnight grown *A. baumannii* AIIMS 7 culture. Direct PCR of total RNA without reverse transcription was used to test for any DNA contamination of RNA samples.

### 2.5. Analysis of MFS-Transporter Like Protein

#### 2.5.1. *In Vitro* and *In Vivo* Translation

*In vitro* translation of *pmt* gene cloned downstream of the SP6 promoter of pGEpmt was analyzed using TNT SP6-High-yield Wheat germ protein expression system (Promega, USA) as per manufacturer's instructions. For comparison, *in vivo* translation was assessed; after overnight culturing in LB broth at 37°C with constant shaking, *E. coli* DH5*α*-pGEpmt and control *E. coli* DH5*α*-pGEM cells were used to prepare whole-cell lysates. Products were analyzed on 17.5% SDS-PAGE stained with Coomassie brilliant blue (0.1%).

### 2.6. Characterization of Clones for Biofilm Formation

#### 2.6.1. Comparative Biofilm Assay of Control and Clones on Abiotic Surfaces (Polystyrene and Glass)

Qualitative biofilm assay on glass tubes was performed as earlier described [25]. Quantitative biofilm assay was performed in 96-well polystyrene microtitre plates [12], and absorbance at 570 nm was read in a Multiplate reader (Molecular Devices, USA). The assays were repeated at least five times. Absorbances were normalized using LB broth as blank.

### 2.7. Quantification of Biofilm Formation on Biotic Surfaces

#### 2.7.1. HeLa Cells

Biofilm formation was quantified as per methods described elsewhere [26] with required modifications. HeLa cells (10^5^ cells/mL) grown in Dulbecco's Modified Eagles Medium (DMEM, HiMedia, India) were seeded in a 96-well microtitre plate and incubated at 37°C in CO_2_ atmosphere for 48 hours. Overnight grown bacterial culture (2 × 10^5^ cells/mL) was resuspended in DMEM and added to the wells followed by incubation for 3 hours at 37°C in CO_2_ atmosphere. The supernatant was aspirated from the wells and washed twice with phosphate buffered saline (PBS) and fixed with 100% methanol. This was followed by staining with 0.1% Giemsa stain and taking absorbance at 590 nm after solubilization in glacial acetic acid.

#### 2.7.2. *Saccharomyces cerevisiae*

 10-hour-old culture of *Saccharomyces cerevisiae* (Baker's yeast) grown in 5% sucrose was inoculated in a 96-well microtitre plate (10^5^ cells/mL) and incubated at 37°C for 12 hours to allow biofilm formation. The supernatant was aspirated, and overnight grown bacterial culture (2 × 10^5^ cells/mL) was added to the wells and incubated at 37°C for 16 hours. This was followed by the quantitative biofilm assay as for polystyrene surfaces [12].

### 2.8. Localization Studies for Adhesion of Clones

#### 2.8.1. Fluorescence Microscopy for Adherence of Clones to Yeast Cells

 In order to observe the adherence of recombinant strains to biotic surfaces (*S. cerevisiae)*, *E. coli* DH5*α*-pGFP: pGEpmt and *E. coli* DH5*α*-pGFP: pGEM were used. Success of cotransformation was verified by plasmid isolation from green-fluorescent colonies observed under UV and positive colony PCR amplification. A sterile slide was placed in a sterile empty Petri dish, and 20 mL of 10-hour-old *S. cerevisiae *culture was added and incubated at 37°C for 12 hours to allow biofilm formation. Excess medium from the plate was discarded, and 20 mL of overnight-grown bacterial culture was added on the *S. cerevisiae* biofilm itself and incubated for 16 hours at 37°C. Supernatant was discarded, and slide was washed thrice with PBS to remove nonadherent cells. The slide was observed at 400x magnification of a fluorescent microscope (Axioscope A.1, Zeiss, Germany).

#### 2.8.2. Scanning Electron Microscopy (SEM) on Abiotic Surfaces (Glass)

 Overnight grown cells (3 × 10^9^ cells) were inoculated on (1 × 1 cm) glass slides inside sterile 12-well culture plates (Tarsons, India) and incubated at 37°C overnight. Culture supernatant was removed, slides were immediately flooded with 2.5% glutaraldehyde in PBS and incubated at room temperature for 2 hours, followed by rinsing with sterile distilled water and serially dehydrated with an ethanol gradient (25–100%), CO_2_ critical point dried and coated with Platinum in a Auto Fine coater (JFC-1600, JEOL, Japan). Coated slides were fixed onto sample holders by carbon tapes and mounted on a scanning electron microscope (Vega, Tescan, USA) with 30 KV input voltage. At least 50 fields were observed at various magnifications and working distances and each sample repeated twice.

### 2.9. Analysis of DNA Release

Purification of extracellular DNA was done from 0.22 *μ*m filtered supernatant of overnight grown cultures of* E. coli* DH5*α*-pGEM, *E. coli* DH5*α*-pGEpmt, and *A. baumannii *AIIMS 7 as per methods described [27]. Briefly, to 750 *μ*L cell-free supernatant (0.22 *μ*m filter sterilized), equal volume of buffer A (50 mM Tris and 10 mM EDTA, 1% CTAB, pH 8.0) was added and incubated at 65°C for 30 min, followed by centrifugation at 8000 rpm for 10 min. To the pellet, 500 *μ*L of buffer B (10 mM Tris, 0.1 mM EDTA and 1 M NaCl, pH 8.0) was added, followed by addition of 0.3 volumes of ice cold isopropanol, incubated for three hours at 4°C, and, finally, pellet was resuspended in 40 *μ*L DNase RNase-free T_10_E_1_ buffer pH~8.0 (Sigma Aldrich). Purified eDNA samples were assessed quantitatively in Biophotometer Plus (Eppendorf, Germany) along with purity and analyzed qualitatively by agarose gel electrophoresis.

#### 2.9.1. Statistical Analysis

Each datum point was averaged from three independent experiments, each with eight replicate wells. All results obtained from nucleic acid quantification and biofilm assays were entered in to excel spreadsheets (Microsoft, USA). Frequency distribution, namely, mean with standard deviations was determined. Statistical analysis was performed by Student's two tailed *t*-tests, *P* value <0.05 was considered to be statistically significant.

## 3. Results

### 3.1. DNA and RNA Manipulations

Amplification and Transcription of the Gene *In vitro* amplification by PCR yielded an amplicon of size 735 bp. To check the transcriptional activity of the gene *in vivo*, RT-PCR was performed using cDNA and an internal primer set (Table 2). The amplified product contained the cDNA regions (246 nt) in the ORF of the gene *pmt* being investigated, and, therefore, it could be confirmed that the gene was actively transcribed in the cells. *DNA Sequencing*. DNA sequencing of the amplicon yielded a 735 nt sequence which was analyzed further to reveal that it contains a ORF of 453 nucleotides. BLASTn results showed 97% identity with the available genome sequences of *A. baumannii* strains AB307-0294, AB0057, AYE (E = 0.0) with specific hits directed towards MFS transporter proteins. The DNA sequence was deposited in GenBank under Accession number HM595762. *Cloning and confirmation*. The purified recombinant plasmid pGEpmt had a size of 3.75 kb. Colony PCR of the transformants showed amplification of the original fragment of ~735 bp indicating successful cloning of *pmt* ORF in the selected colonies (data not shown).

### 3.2. Differential Expression of *pmt* Gene

**Figure 1:**
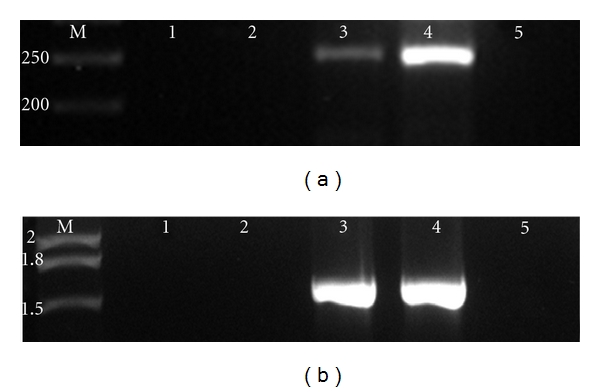
Differential expression of *pmt* of *A. baumannii* AIIMS 7 (a) expression of *pmt.* Lane 1: direct PCR using total RNA from planktonic cells (nonadherent) of *A. baumannii* AIIMS 7; Lane 2: direct PCR using total RNA from biofilm cells (adherent) of *A. baumannii* AIIMS 7; Lane 3: RT-PCR using total RNA from planktonic cells (nonadherent) of *A. baumannii* AIIMS 7. Lane 4: RT-PCR using from biofilm cells (adherent) of *A. baumannii* AIIMS 7 cells (b) Expression of internal control 16SrDNA gene. Lane 1: direct PCR using total RNA from planktonic cells (nonadherent) of *A. baumannii* AIIMS 7; Lane 2: direct PCR using total RNA from biofilm cells (adherent) of *A. baumannii* AIIMS 7; Lane 3: RT-PCR using total RNA from planktonic cells (nonadherent) of *A. baumannii* AIIMS 7; Lane 4: RT-PCR using from biofilm cells (adherent) of *A. baumannii* AIIMS 7 cells.

Significantly higher expression of the *pmt* was observed in adherent cells of biofilm (scraped cells from bottom), as compared to the planktonic cells (nonattached) (Figure 1(a)). We intended to evaluate the level of transcription upon adherence on to surface; the observations as indicated by the intensity (Figure 1(a)) denote a high degree of transcription of this region of the ORF and demonstrate direct evidence of functionality of this gene in biofilm mode of growth. The negative controls showed no amplification, indicating absence of DNA in the sample (Figure 1(a), lane 1, 2). *16SrRNA* gene was amplified as negative control (Figure 1(b)).

### 3.3. Analysis of Protein

**Figure 2:**
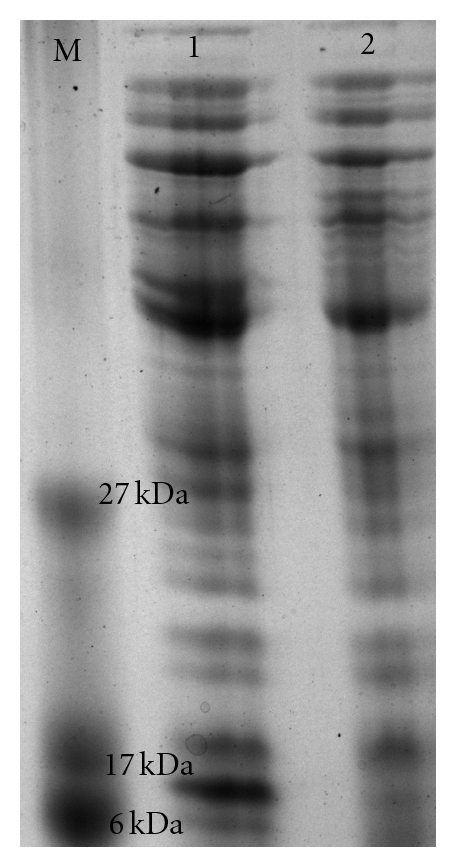
*In vitro* translated protein products. Lane M: ultra low-range peptide molecular weight marker, Lane 2: product of *in vitro* translation of pGEpmt, Lane 3: negative control (*E. coli* DH5*α*-pGEM).

**Figure 3:**
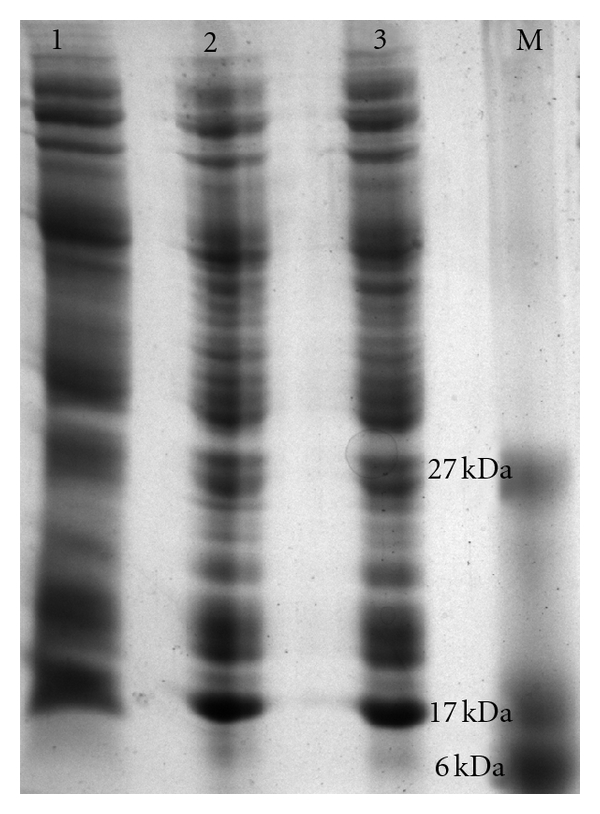
Total protein profile of recombinant *E. coli DH5*α** showing *in vivo* translation. Lane 1: total protein profile of *E. coli* DH5*α*-pGEM, Lane 2: total protein profile of *E. coli* DH5*α*-pGEpmt, Lane 3: total protein profile of *E. coli* DH5*α*-pGEpmt after IPTG induction.

Using the obtained nucleotide sequence, the ORF yielded 150 amino acid residues by theoretical translation. The obtained ORF was much shorter than predicted MFS transporter proteins (400 amino acid residues) present in the genomes of *A. baumannii* typed strains. BLASTp analysis of the amino acid sequence showed presence of conserved motifs specific to MFS transporter superfamily proteins with high similarity (up to 72%). Prediction of transmembrane helices in proteins using TMHMM Server v. 2.0 indicated presence of two transmembrane helices (amino acid position: 67–89; 93–115). PROSITE pattern analysis indicated that the entire ORF was a signature sequence of MFS transporter superfamily. *In silico* proteomic feature analysis (ExPASy tools, ProtParam) predicted a translation of Pmt protein of ~16.95 kDa with an Isoelectric point (pI) of 9.67. When we evaluated this using the *in vitro* translation experimentally, a distinctly overexpressed band of ~17 kDa appeared on SDS-PAGE and (Figure 2) indicated that and was in accordance with the *in silico* prediction. Moreover, total protein profile analysed from clones also showed the overexpressed protein of same size (~17 kDa) on induction with IPTG (Figure 3, Lane 3). Our analysis suggested that presence of transmembrane helices in the Pmt protein might enable it to function as a transporter protein in *A. baumannii* AIIMS 7.

### 3.4. Characterization of Adherence and Biofilm Formation

#### 3.4.1. Comparative Biofilm Assay of Control and Clones on Abiotic Surfaces (Glass and Polystyrene)

**Figure 4:**
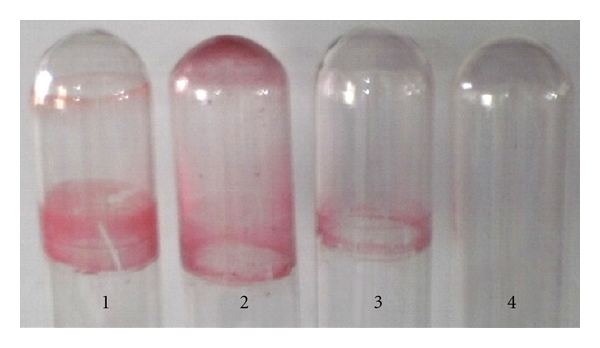
Qualitative assay showing biofilm formation on abiotic (glass) surface. biofilms formed by 1: *A. baumannii* AIIMS 7, 2: *E. coli* DH5*α*-pGEpmt, 3: *E. coli* DH5*α*-pGEM, 4: negative control (LB broth).

**Figure 5:**
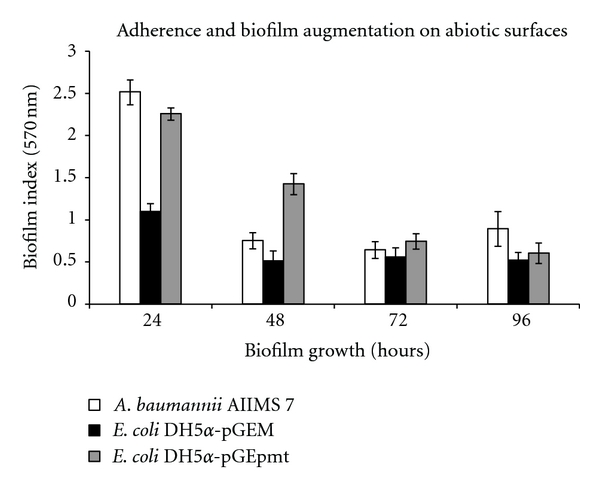
Quantitative analysis of adherence and biofilm formation on abiotic surface (polystyrene microtitre). (Absorbance values normalized with blank medium, *P* < 0.05.)

Surface adhesion and biofilm formation were tested on model surfaces that are clinically important. With due consideration on the ability of *A. baumannii *to persist on inanimate surface in clinical environments as well as inside the host tissue by forming biofilms, two surfaces of each abiotic and biotic were selected. Qualitative biofilm assay performed using a simple assay (glass tube method) showed direct biofilm formation and augmentation on glass surfaces in comparison to control *E*. *coli *DH5*α*-pGEM (Figure 4). On 96-well polystyrene surface, quantitative assay showed marked biofilm augmentation by the *pmt* clones. A temporal evaluation of the same was assessed till 96 hours. Results showed maximum expression of gene in the beginning stages of biofilm formation (24 hour and 48 hour; 2.05- and 2.79-fold increase respective); 2.79-, 1.33-, 1.16-fold increase, respectively, at 72 and 96 hours (Figure 5). This also correlated well with our differential expression by RT-PCR assay where biofilm or attached cells showed enhanced transcription of the gene.

#### 3.4.2. Quantification of Biofilm Formation on Biotic Surfaces (HeLa Cell Line and Yeast Cells)

 Similar to abiotic surface, representative biotic surfaces were also chosen.

**Figure 6:**
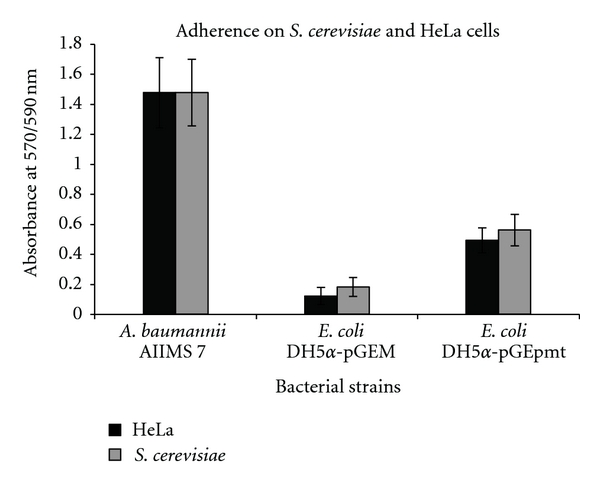
Quantitative analysis of adherence and biofilm formation on biotic surfaces (*S. cerevisiae* and HeLa cells). (Absorbance values normalized with blank medium, *P* < 0.05.)

HeLa Cells. Significant increase in adherence onto human (HeLa cell line) cells was observed for the *E*. *coli *DH5*α*-pGEpmt. 4.1-fold increase in adherence was observed as compared to control *E*. *coli *DH5*α*-pGEM (Figure 6, black bars). *S. cerevisiae*. A 3.1-fold increased adherence was observed in *E*. *coli *DH5*α*-pGEpmt as compared to control *E*. *coli *DH5*α*-pGEM as was observed (Figure 6, grey bars) in case of the recombinants containing the gene.

### 3.5. Localization of Adherence

#### 3.5.1. Fluorescence Microscopy for Adherence of Clones to Saccharomyces  cerevisiae Yeast Cells

**Figure 7:**
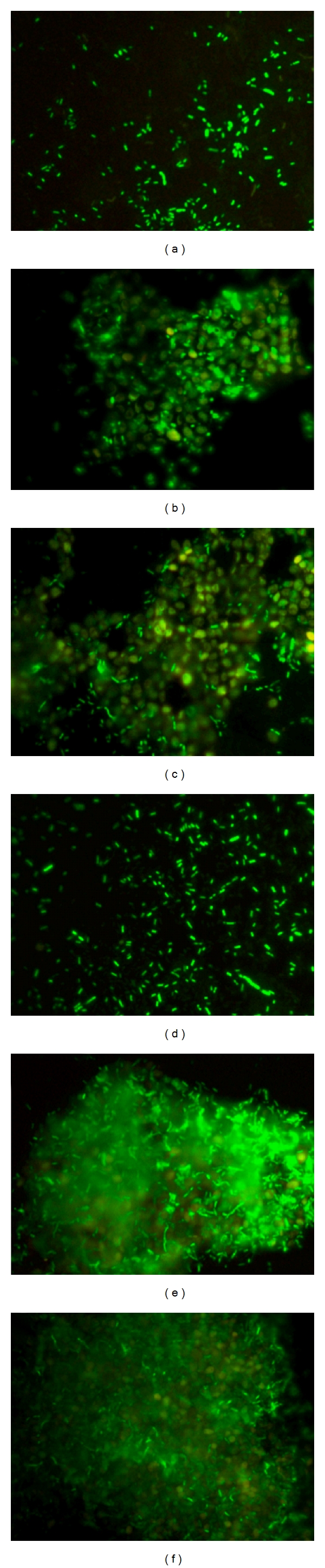
Fluorescence microscopic analysis of adherence to *S. cerevisiae*. (a) *E. coli* DH5*α*-pGFP: pGEM cells. (d) *E. coli* DH5*α*-pGFP: pGEpmt cells. (b), (c) Adherence of control (*E. coli* DH5*α*-pGFP: pGEM) cells to *S. cerevisiae* cell surface beneath. (e), (f) Adherence of* E. coli* DH5*α*-pGFP: pGEpmt cells on *S. cerevisiae*. Intense green fluorescence indicates expression of *gfp*-tag, whereas *S. cerevisiae *cells are seen as yellow colored. (Images taken at 400x.)

 To assess the adherence capabilities augmented due to expression of the protein, we performed the *gfp*-tagged fluorescence microscopy. When compared to the control cells, the clones exhibited marked increase in the adherence to yeast cells (Figure 7). The primary biofilm formed by *S. cerevisiae* was almost completely colonized by biofilm formed by the recombinants. The EPS formed by the bacteria could be seen prominently as hazy layers (Figure 7(f)), covering biofilm of *S. cerevisiae.* The number of adhering bacterial cells on the surface of *S. cerevisiae* biofilm was clearly much larger in case of recombinants.

#### 3.5.2. Scanning Electron Microscopy

**Figure 8:**
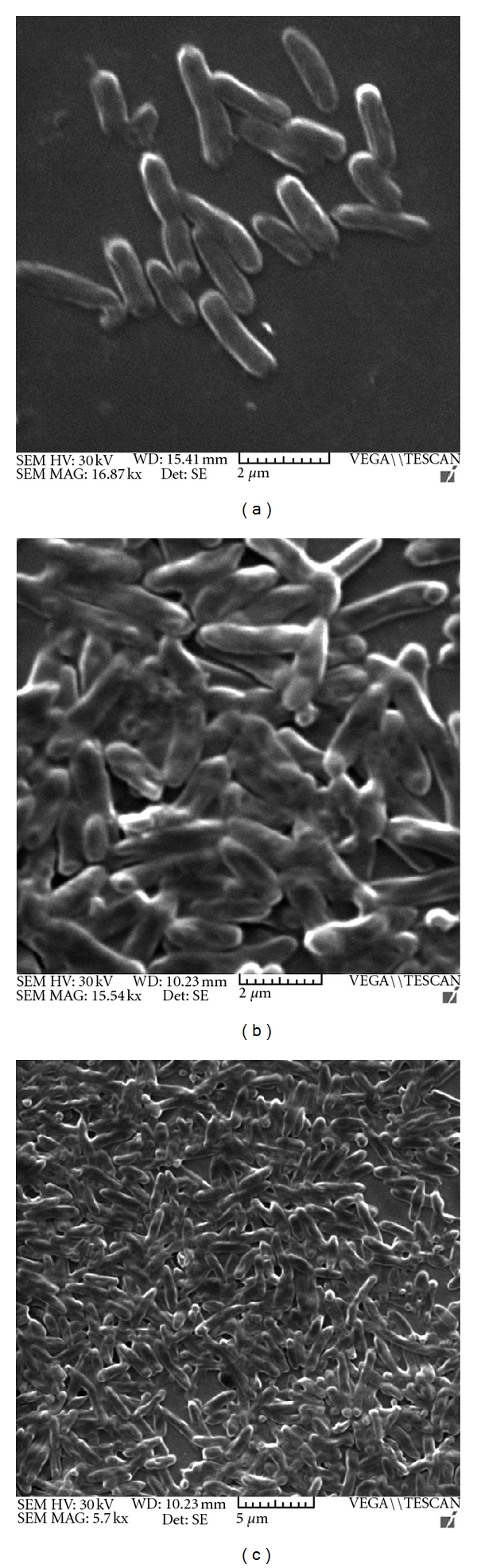
Scanning electron microscopy images showing adherence and biofilm formation on glass. (a) *E. coli* DH5*α*-pGEM cells adhering to abiotic (glass) surface. (b), (c) *E. coli* DH5*α*-pGEpmt cells adhering together and forming biofilm on glass surface. (Bars and magnification indicated below representative figures.)

 To address the question of enhancement of adherence on to abiotic surface, SEM was performed, where representative micrographs showed significantly dense and thicker biofilm formed by the *E*. *coli *DH5*α*-pGEpmt (Figures 8(b) and 8(c)) as compared to control *E*.*coli *DH5*α*-pGEM (Figure 8(a)). 

#### 3.5.3. Analysis of eDNA

**Figure 9:**
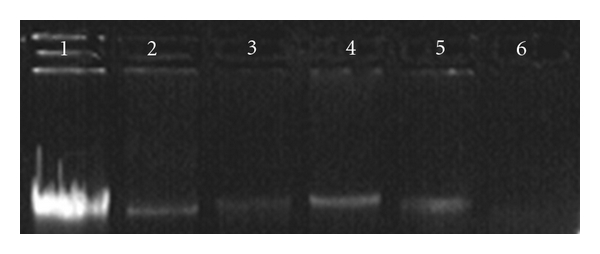
Qualitative analysis of eDNA production. Lane 1: gDNA of *A. baumannii* AIIMS 7 (Positive control), Lane 2: eDNA produced by *E. coli* DH5*α*-pGEpmt, eDNA, Lane 3: eDNA produced by *E. coli* DH5*α*-pGEM, Lane 4-5: eDNA produced by *A. baumannii* AIIMS 7, Lane 5: negative control (Luria broth).

**Figure 10:**
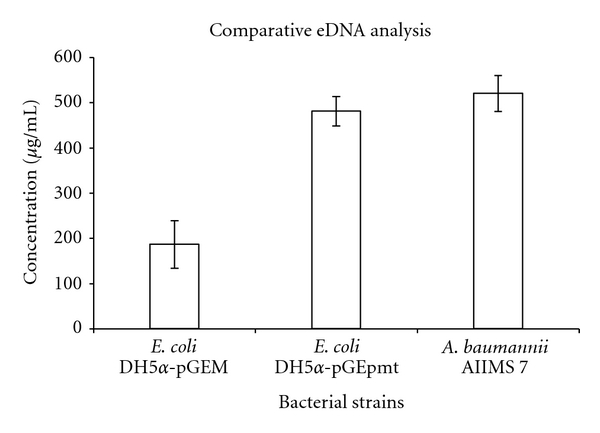
Quantitative analysis of eDNA production during *in vitro* growth.

Figure 9 shows the eDNA purified and analysed on agarose gels; distinct presence of equivalent molecular weight eDNA from the clones can be seen with Lane 1 showing control genomic DNA from *A. baumannii *AIIMS 7. When quantitatively analyzed, as shown in Figure 10, about 2.58-fold increase in eDNA production was seen in *E. coli* DH5*α*-pGEpmt (480.7 ± 52.69 *μ*g/mL) as compared to the *E*. *coli *DH5*α*-pGEM (186.5 ± 32.52 *μ*g/mL). eDNA concentration in *A. baumannii* AIIMS 7 was found to be 520.0 ± 40.0 *μ*g/mL.

## 4. Discussion

Members of at least three types of transport systems, namely, ATP-binding cassette (ABC)-type, resistance nodulation division (RND)-type, and major facilitator superfamily (MFS)-type transporters, have been proposed so far, to function together with trans-periplasmic proteins called membrane fusion proteins (MFP) to facilitate transport across both membranes of the Gram-negative bacterial cell envelope [28]. The MFS consists of membrane transport proteins which are found in several bacteria to higher eukaryotes and are involved in the symport, antiport, or uniport of various substrates, such as sugars, Krebs cycle intermediates, phosphate esters, oligosaccharides, and antibiotics [29]. It is known that members of the MFS transporter family could drive the export of cytoplasmically derived molecules, namely, DNA across the two membranes of the Gram-negative bacterial cell into the external milieu.

It was hypothesized that the characterized MFS transporter-like protein has a similar role of transport with greater implication; especially with reference to emerging pathogens like MDR *A. baumannii,* which is known to devote considerable portion of its genes to pathogenicity [30]. We characterized this gene *pmt* in an highly MDR pathogenic *A. baumannii *AIIMS 7; whose over-expression of the ~17 kDa protein showed marked increase in cellular adherence as well as biofilm augmentation. Moreover, the sequence homology to “putative MFS transporter proteins” of *Acinetobacter baumannii* indicated with larger probability that it could be involved in nucleic acid transport. Our observation also brings to light a probable function of this transporter gene to be involved in nucleic acid export as indicated by analysis of eDNA release (Figures 9 and 10). The function of eDNA release was thought to be due to uncommon evolution (with regard to pathogenicity) of *A. baumannii* from close members of the genus (which are naturally competent) and the adaptability of its genome to perform function like spreading resistance genetic traits through eDNA owing to its ubiquity in nature and survival. In our recent work [22], we have characterized the role of eDNA in biofilm formation as well as augmentation on abiotic surface in *A. baumannii* AIIMS 7. Although presence of eDNA in the extracellular growth medium may be contributed by minimal amount of passively released DNA from cell lysis at later stages, significant amount of increased eDNA content from *pmt* clones under similar growth conditions as in the control cells may well justify our current hypothesis that MFS transporter-like proteins could be involved in eDNA release.

As reviewed earlier [31], developmental progression of surface-attached bacterial communities would require differential expression of various genes. Downregulation of flagellar biosynthetic machinery upon surface attachment in *P. aeruginosa *[32] and biofilm-dependent gene regulation in *E. coli *[33] has been studied. In *A. baumannii *we have shown overexpression of *pmt* gene in 24-hour biofilm bacteria (Figure 1), than in nonadherent and/or planktonic bacteria indicating that the expression is *surface induced* and *stage specific*. SEM and fluorescence microscopic analysis (Figures 7 and 8) also supported this finding, as the phenotypic expression in terms of increased adherence, was seen only at surfaces. Interestingly, RT-PCR analysis from 24-hour old biofilm showed high expression also correlated well with our quantitative biofilm assay (Figure 5) showing maximum biofilm at 24 hours and gradually decreasing till 96 hours. Specificity of gene expression being surface and stage specific may correlate to important parameters in severity of the infection, for example, expression of such genes would largely affect the stability as well as infectious nature of biofilms in clinically important surfaces as urinary catheters in patients with urinary tract infections, as shown recently in our study [6]. However, biofilm augmentation due to Pmt (putative MFS transporter-like gene) expression, in general, would be in coordination with various other determinants of biofilm formation like macromolecular secretions (EPS matrix and nucleic acids), biofilm associated proteins, autolysin proteins, and so forth [34]. The collective expression of these determinants would account for the extremely strong biofilm formation by *A. baumannii *[35] and may be part of a tight regulation. After cloning of *pmt *in *E. coli*, 2.1-fold increase in biofilm formation on abiotic (polystyrene surfaces) was observed as compared to control *E. coli* and substantial increase in biofilm formation on glass was observed. Maximum biofilm augmentation on polystyrene surfaces was observed at 24 hours of growth (corresponding to stationary phase in the growth of bacteria). A 4.1-fold increase in adherence to biotic surfaces (HeLa cell line) was observed in case of *E. coli* DH5*α*-pGEpmt. A novel approach was used to detect the adherence of the recombinants with pGEpmt plasmid by gfp-tagged fluorescence microscopy. Adherence of these recombinants on *S. cerevisiae* was studied using *E. coli*-pGFP: pGEM as a control. Significant increase in attachment of clones to *S. cerevisiae* cells as compared to *E. coli*-pGFP: pGEM (Figure 6) was seen, indicating the role of *pmt* in adherence to biotic surfaces presenting a “*preferred choice*” of surface, that is, biotic over abiotic. 

Taken together, this work depicts the characterization of a newly identified MFS transporter-like gene *pmt*, from a MDR clinical isolate of *A. baumannii *isolated from an ICU in India. First, it shows differential expression in biofilm mode, which largely implicates its role in adherence and biofilm formation, with the expression being stage dependent. This was further assessed using *E. coli* model of heterologous expression; specifically the characterization of adherence and biofilm augmentation on chosen biotic and abiotic surfaces. Furthermore, the strong sequence homology of *pmt* ORF to MFS transporter proteins, presence of transmembrane helices in the protein and eDNA analysis, it may be associated with eDNA release in *A. baumannii, *although further studies are warranted to establish this function. Nevertheless, studies on transporter proteins of the ubiquitous bacterium of *Acinetobacter *genus with role(s) in biofilm formation on biotic and abiotic surfaces might fascinate microbiologists more, not only because of the diverse implication of MFS superfamily of proteins in multiple molecular, physiological, and metabolic processes, but also the uniqueness of this genus in being involved in natural competence, genomic diversity, multidrug resistance, gene transfer, pathogenesis, and biofilm formation. Especially structure, function, and phylogeny of the array of uncharacterized transporter proteins in *MDRAB* may provide possibilities for development of new strategies to inhibit or modify binding of pathogens to clinically important as well as host surface, providing effective therapeutic options for combating biofilm pathogenesis.

## Figures and Tables

**Table 1:** Strains and plasmids used in the study.

Description	Characteristics	Reference
*Acinetobacter baumannii *AIIMS 7	Clinical isolate from neurosurgery ward, All India Institute of Medical Sciences (AIIMS), New Delhi, India	[23]
*E. coli*		
DH5*α*	Used for all cloning and recombinant DNA methods	Invitrogen
pGEpmt	*E. coli* harbouring *pmt* gene cloned into pGEMT-Easy vector	This study
pGEM	*E. coli *harbouring control plasmid pGEM3zf (+)	This study
pGFP: pGEpmt	pGEpmt and pGFP cotransformed in *E. coli *DH5*α*	This study
pGFP: pGEM	pGEM3zf (+) and pGFP cotransformed in *E. coli *DH5*α*	This study
Plasmids		
pGEMT-Easy	Used to clone *pmt *	Promega
pGFP	Used for *gfp* tag	ClonTech, Takara

**Table 2:** Oligonucleotides used in the study.

Details	Primer name	5′-3′ sequence	Product length	Reference
Direct PCR primers	pmt-F	TAGGGGTATCACCATTTGTG	735 bp	Present study
	pmt-R	TCATGTATAACGACACCAGT		
RT-PCR primers	pmt-RT F	AGTCTTTGGCCATTTTGGTG	246 bp	Present study
	pmt-RT R	GCAACTTCCCAAACCCTGTA		
16S rRNA	16S F	TGGCTCAGATTGAACGCTGGCGGC	1500 bp	Lee et al., 2008 [12]
	16S R	TACCTTGTTACGACTTCACCCCA		
